# Draft Genome Sequence of Biological N_2_-Fixing Bacterium Rhizobium tropici A12, Isolated from Root Nodule of Tropical Soybean (Glycine max), TGx-1148 Variety

**DOI:** 10.1128/MRA.00189-20

**Published:** 2020-05-14

**Authors:** Slimane Khayi, Rachid Mentag, Martin Jemo

**Affiliations:** a Biotechnology Unit, Regional Center of Agricultural Research of Rabat, National Institute of Agricultural Research, Rabat, Morocco; b AgroBiosciences Program, Mohammed VI Polytechnic University (UM6P), Ben Guerir, Morocco; University of Arizona

## Abstract

Here, we present the genome sequence and annotated features of Rhizobium tropici strain A12, which is able to nodulate tropical Glycine max (soybean). The draft genome of the strain consists of 107 contigs totaling 6.5 Mbp in size. The annotation revealed 6,204 protein-coding genes, 52 tRNAs, and 7 rRNAs.

## ANNOUNCEMENT

The genus *Rhizobium* includes several species of soil bacteria with abilities to fix atmospheric dinitrogen (N_2_) through an endosymbiotic association with roots of legumes (1). These bacteria harbor genes responsible for the conversion of atmospheric N_2_ into organic nitrogenous compounds (ammonium [NH_4_^+^]) able to be assimilated by plants. Through the nitrogen fixation process, legumes play vital roles in cropping cycles through soil fertility restoration, as well as serving for food and income generation for small farmers (2). The species Rhizobium tropici is considered the most efficient symbiont of Phaseolus vulgaris (common bean) in tropical acid soils; it is also very promiscuous and is able to establish symbiotic associations with a variety of legumes (3–5). Here, we present the draft genome sequence of R. tropici strain A12, which was isolated from tropical Glycine max plants under African soil conditions.

**FIG 1:**
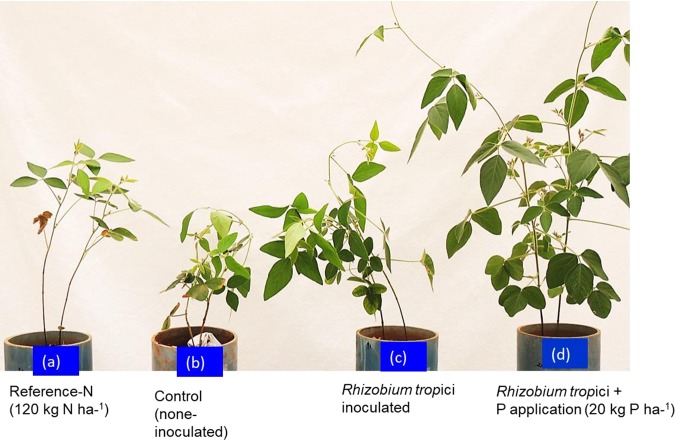
Growth and development of soybean variety TGx-1148 at 8 weeks after sowing, with reference nitrogen application (120 kg N ha^−1^) (a), no rhizobium inoculation (b), R. tropici A12 inoculation (c), or R. tropici A12 inoculation plus P application at 20 kg P ha^−1^, as triple superphosphate (d).

The rhizobia were isolated from root nodules of soybean plants grown for 8 weeks under greenhouse conditions. The strains were morphologically identified, and fresh pure cultures were preserved in the International Institute of Tropical Agriculture (IITA) Genebank. R. tropici strain A12 was cultured on yeast extract-mannitol-broth (YMB) medium (yeast extract, 1 g/liter; mannitol, 10 g/liter; dipotassium phosphate, 0.5 g/liter; magnesium sulfate, 0.2 g/liter; sodium chloride, 0.1 g/liter; calcium carbonate, 1 g/liter) at 28°C, and 2 ml of fresh culture was used to inoculate a 5-day-pregerminated soybean cultivar TGx-1148 seedling. The experiment’s design has four treatments, (i) reference N application at 120 kg N ha^−1^, (ii) control with no inoculation, (iii) R. tropici A12 inoculation, and (iv) R. tropici A12 inoculation plus P application at 20 kg P ha^−1^ (Fig. 1).

Genomic DNA extraction was performed from an overnight culture of a single colony on YMB medium using the Qiagen DNA purification kit. Quantiﬁcation and quality control of the DNA were completed using a NanoDrop ND1000 device and 1.0% agarose gel electrophoresis, respectively. The genomic DNA was subjected to next-generation sequencing technology. A paired-end library was constructed using the NEBNext Ultra DNA library preparation kit for Illumina (New England Biolabs, United Kingdom) and sequenced on a MiSeq system (Illumina, San Diego, CA, USA) using the PE-250 module, yielding 1,871,679 reads (Biosciences eastern and central Africa-International Livestock Research Institute, Nairobi, Kenya). After DNA sequence quality trimming and adaptor removal using the BBMap suite (https://github.com/BioInfoTools/BBMap), 1,648,214 clean reads were obtained, corresponding to 289,620,715 bases. *De novo* assembly was performed using SPAdes software (v3.11.1) with default parameters (6). The assembly generated 107 contigs (>2,000 bp) totaling 6,590,945 bp with an average coverage of 44×. The average length of the contigs was 61,597 bp, and the largest contig was 391,517 bp, with a GC content of 59.55% and a contig *N*_50_ value of 119,397 bp. The genome sequence of R. tropici A12 was annotated using the NCBI Prokaryotic Genome Annotation Pipeline (PGAP) (7), which resulted in a total of 6,204 protein-coding genes, 52 tRNAs, and 7 rRNAs.

### Data availability.

This whole-genome shotgun project has been deposited at DDBJ/ENA/GenBank under accession number JAADZA000000000. The version described in this paper is the first version, JAADZA010000000. The Illumina reads are available in the SRA under accession number SRX7775460.
